# Inferring Novel Indications of Approved Drugs via a Learning Method with Local and Global Consistency

**DOI:** 10.1371/journal.pone.0107100

**Published:** 2014-09-30

**Authors:** Yan Yan, Xinwei Shao, Zhenran Jiang

**Affiliations:** 1 Department of Computer Science & Technology, East China Normal University, Shanghai, China; 2 Department of Mathematics, East China Normal University, Shanghai, China; University of Sassari, Italy

## Abstract

Inferring new indication of approved drugs is critical not only for the elucidation of the interaction mechanisms between these drugs and their associated diseases, but also for the development of drug therapy for various human diseases. This paper proposes a network-based approach to reveal the association between 52 human diseases and potential therapeutic drugs based on multiple types of data. The advantage of the approach is that it can obtain the global relevance features for each drug-disease pair in the network by the learning local and global consistency method (LLGC). Cross-validation tests results demonstrate the proposed approach can achieve better performance comparing with previous methods. More importantly, it provides a promising strategy to maximize the value of therapeutic drugs and offer safe and effective treatments for different diseases.

## Introduction

The traditional mode of drug development is a long and costly process. The investigation shows that it requires an investment of about $1 billion dollars and takes about 10∼15 years to take a compound from discovery to the approved medication [1]. In fact, more than 90% of the drugs have been proved ineffective before being clinically tested [2]. In order to improve the productivity of drug discovery, there is an urgent need for developing computational methods to address new treatment opportunities by utilizing the available information of known drugs. Undoubtedly, inferring novel indications of approved drugs is an effective way to achieve this important goal.

Previous studies have demonstrated that genome-wide transcriptional expression data is useful to interpret biological influence of drugs or disease states [3], there has been a trend for drug-disease study based solely on the analysis of gene expression data over the past years. The popular methods including Gene Enrichment Analysis method (GSEA) and the Connectivity Map (cMap) have proved effective in discovering new therapeutic drugs for some diseases [4]. Both of the methods are based on the hypothesis that if two diseases share similar therapies, then the drug used for this disease may also be therapeutic for other diseases [5]. For instance, GSEA can be used to determine whether a priori defined set of genes shows statistically significant, concordant differences between two biological states [6]. cMap is often used to seek the functional associations between drug response profiles and diseases through the transitory feature of common gene-expression changes [7]. However, the methods are greatly affected by the quality of gene expression data due to the profiles generated under different conditions. Therefore, it is incapability to capture drug-disease associations that are not manifested on the gene expression level.

Recently, some researchers attempted to incorporate the biological and chemical knowledge to construct effective feature vectors of drug-disease pairs with different learning methods. For instance, Li et al. [8] proposed a literature mining method to build disease-specific drug protein cMaps via protein interaction networks. Gottlieb et al. [9] attempted to predict new drug indications by different feature information including gene expression profile, chemical structure, side effects and chemical protein interactome. Nevertheless, few researches have attempted to maximize the information flow in the disease network for uncovering new drug-disease pairs. Further, the similarity measure methods they used were limited and did not adjust the algorithm according to the specific situations of the samples.

Here, we propose a method by learning with local and global consistency (LLGC) to predict new drug-disease associations [10]. For each drug-disease pair in a network, we obtain the feature vectors of 609 known drug-disease associations based on different types of data. Compared with the PREDICT method, our method can obtain a higher specificity and sensitivity. Further, we expect the top-scored drug-disease pairs highly enriched in literature can be used for further clinical trial.

## Materials and Methods

### Data sets

In this work, we first collect 42 kinds of drugs associated with breast cancer from KEGG database [11]. Then we obtain other 51 kinds of diseases with which the 42 drugs are associated. These 52 diseases totally have 609 known associations with 203 FDA approved drugs. Since each selected disease has at least one public associated drug with breast cancer, drugs treating one or several of these 51 diseases may also has some effect on treating breast cancer [7]. Finally, we got 609 drug-disease pairs and 668 drug-target interaction pairs (192 targets corresponding to these 203 drugs) in the disease network.

### Overview of the method

**Figure 1:**
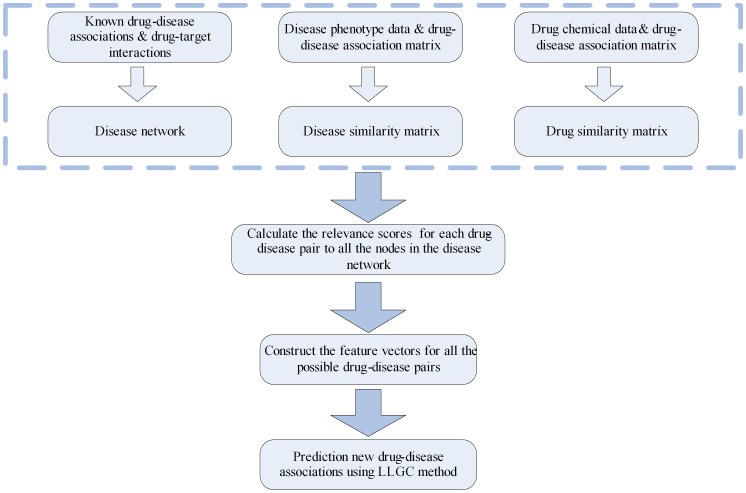
The flowchart of the prediction method.

According to the LLGC method, we first constructed the disease-drug networks based on multiple types of feature information. Then, the feature vectors for all of the possible drug-disease pairs were obtained based on LLGC methods. Finally, new possible drug-disease associations can be predicted based on the analysis of network topology. Figure 1 shows an illustration of the above procedure.

### Similarity measures

In the paper, four similarity measures were used to obtain results that are more reasonable.

1. **Chemical structure similarity.** SIMCOMP [12] is an online tool provided by the KEGG LIGAND database (http://www.genome.jp/ligand), which offers a global similarity score by the ratio between the size of common sub-structures and the size of the union structures of two compounds. The similarity between two compounds c and c′ is usually computed as follows:

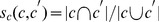
(1)


SIMCOMP can only calculate the compounds whose similar scores are greater than a certain threshold. Therefore, we used the SIMCOMP2 (http://www.genome.jp/tools/simcomp2/) to obtain the chemical structure similarity between any two compounds in order to construct a similarity matrix denoted 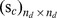
(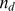 is the number of drugs).

2. **Phenotypic similarity.** We used the phenotypic similarity constructed by van Driel et al. (2006) [13]. The phenotypic similarity 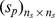 was constructed by identifying the similarity between MeSH terms [14] appearing in the medical description of diseases from OMIM database [15] (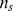 is the number of diseases).
3. **Network topology information.** In our study, we constructed an adjacency matrix to represent all of the known drug-disease associations. The underlying assumption here is that if two drugs (diseases) share more diseases (drugs), they are more similar. If a drug associates with a disease, the corresponding element was set a value 1. Otherwise, it was set a value 0. We defined two sharing degree similarity matrixes 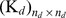 and 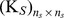 based on Xia et al. (2010) [16]. The element in the 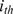 row and 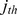 column of 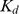 (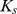) represents the number of diseases shared by the 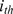 drug (disease) and the 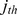 drug (disease). Meanwhile, Floyd algorithm was used to seek the shortest paths between any two nodes in the graph M4 (has been described in Network topology features). For the 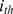 drug, we obtain a vector 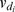 constituted of the lengths between the 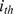 drug and all of the diseases. Then we compute the Pearson correlation coefficient between the vectors of any two drugs to construct a similarity matrix 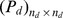 which reflects the topology similarities of drugs. Similarly, we construct a similarity matrix 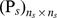 to reflect the topology similarities of diseases.

Pearson correlation coefficient is used to measure the degree of correlation between two variables *X* and *Y*, which is described as follows:
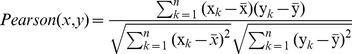
(2)


Obviously, the absolute value of it is bigger, the degree of correlation between *X* and *Y* is higher. For two drugs 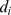, 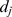 the shortest path similarity of them is computed as follows:
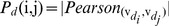
(3)


Based on the method of Perlman et al. (2011) [17], we combine two similarities including the sharing degree similarity and the shortest path similarity to a single topology similarity by computing their weighted geometric mean. In this way, we obtain the topology similarity matrix 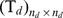 and 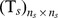.

4. **Combination of the biology similarity and the topology similarity.** The drug similarity matrix can be obtained by the linear combination 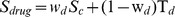 based on Chen et al (2012) [18]. Similarly, the disease similarity matrix can be obtained by 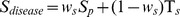. Here, the parameter 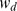 and 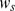 represent the weight of biology similarity evaluation in the integrated similarity measure.

### Construction of graph relevance vectors

The relevance between a drug D and a disease node in the disease network depends on whether the drug has an association with the disease. If the drug has an association with the disease, the relevance score is 1, otherwise the relevance score is 0. We can get the relevance score between disease S and a drug node in the disease network. The calculation process of the relevance score between a drug D (or a disease S) with a node in the disease network is introduced as follows:

- We used the relevance score between drug D and other drug node to describe the drug similarity. Further, we used the relevance between disease S and a disease node to describe the disease similarity. If drug D and a target has known interaction, the relevance score is 1, otherwise, the relevance score is the maximum value of the similarities between drug D and drugs with known interactions with the target.
- We used the relevance score between disease S and a target node in the disease network to describe the maximum value of the relevance scores between the target and all of the drugs with associations with disease S.

### Construction of the feature vectors

The graph relevance vector of the drug-disease pair <*d*, *s*> is 447-dimension vectors. Then we calculate the similarity of the graph relevance vector and obtain 609-dimension feature vectors of each drug-disease pair based on Laarhoven et al. (2011) [19]. The similarity between the graph relevance vector of <*d*, *s*> and the graph relevance of a known drug-disease pair can be computed as follows:
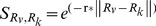
(4)


Where 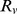 is the graph relevance vector of <*d*, *s*>, 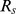 is the graph relevance vector of a known drug-disease pair, *r* is the control parameter:
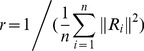
(5)


Where n is the number of the known drug-disease associations, 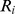 is the graph relevance vector of a known drug-disease pair.

### Computation of network topology features

In order to investigate the network topology features of the disease network, we first embedded four kinds of relationship networks (drug-disease network, drug-drug network, drug-target network, target-target network) into one integrated network. Then, seven types of topological features were used to analyze the topological structures [20]–[21]. The topological features are introduced as follows, respectively.

#### (1) Network diameter

The network diameter D refers to the maximum value of the shortest path lengths of between all nodes in the graph G, which is computed as follows:
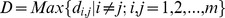
(6)


*d_i_*_,*j*_ refers to the length of the shortest path between *v_i_* and *v_j_*, *m* is the number of nodes. If the network is an unconnected graph, we use its maximal connected sub-graph replace of using infinity to describe the network diameter.

#### (2) The characteristic path length

The characteristic path length refers to the mean value of the shortest path lengths among all nodes in the graph G, which is computed as follows:
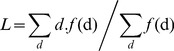
(7)


*F(d)* refers to the frequency that the shortest path length *d* appears. If the network is an unconnected graph, its characteristic path length represents the mean path lengths of the nodes.

#### (3) Network connectivity efficiency

Network connectivity efficiency *E* is an index describing the degree of separation of nodes in the network. *E* is bigger means the connectivity of the network is better. *E* is described as follows:
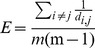
(8)


#### (4) The average degree of network

The average degree of network *k_v_* is the mean value of the degrees of all nodes in the network, which is computed as follows:
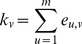
(9)


#### (5) The coefficient of variation

The coefficient of variationξdescribes the extent of network heterogeneity, which is computed as follows:
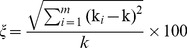
(10)


*k_i_* is the degree of node *v_i_, k* is the mean degree of all the nodes.

#### (6) Clustering coefficient

*k_v_* is the degree of node *v, E_v_* is the number of edges among the adjacent *k_v_* nodes, then the clustering coefficient of node v is:
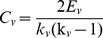
(11)


The clustering coefficient of the network represents the average value of the clustering coefficients of all the nodes, which describes the extent of relationships among the adjacent nodes.

#### (7) Network structure entropy

Entropy is a measure of the uniformity of the energy distribution. Higher entropy means the message contain more information and vice versa. The calculation process of network structure entropy is described as follows:

For the undirected graph G that has m nodes, *k_i_* is the degree of node *v_i_*, then the importance of *I_i_* is *v_i_*:
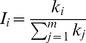
(12)


The network structure entropy of the undirected graph is described as:
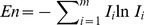
(13)


When the network is completely uniform, *I_i_* = 1/*m*, the maximal value 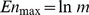; When the network is star-like, the network is more uneven. This moment, the network structure entropy is minimum and has the minimal value *En_min_* 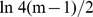.

In order to eliminate the effect of the number of nodes on the entropy, we attempt to normalize the network structure entropy. The normalized network structure entropy is computed as:
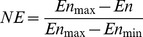
(14)


## Results and Discussion

### Network topology and reliability analysis

#### (1) Analysis of network topology features

*B* refers to the number of edges in the network, *D* refers to the network diameter, *L* refers to the characteristic path length, *E* refers to the network connectivity efficiency, *K* refers to the average degree of the network, *C* refers to the clustering coefficient, *NE* refers to the standard network structure entropy,*ξ* refers to the characteristic value of the variation coefficient of degree, *M*_1_ refers to the drug-disease network, *M*_2_ refers to a network including *M*_1_ and the drug-drug network, *M*_3_ refers to a network including *M*_2_ and the drug-target network, *M*_4_ refers to a network including *M*_3_ and the target-target network. Table 1 lists the feature information used in the disease network.

**Table 1:** Topology features for each network.

	*B*	*D*	*L*	*E*	*K*	*C*	*NE*	*ξ*
***M*** **_1_**	609	6	3.828	0.208	4.776	0	0.275	2001.357
***M*** **_2_**	4772	5	2.303	0.305	37.235	0.352	0.245	1659.563
***M*** **_3_**	5381	7	3.005	0.384	24.217	0.515	0.340	3041.565
***M*** **_4_**	5476	7	2.946	0.390	24.642	0.542	0.323	2946.382

As we can see from **Table 1**, the variation coefficient of degree*ξ*in these networks are high, indicating that the networks are all considerable heterogeneous (meaning that only a few nodes in the network have a large number of connections, most of the nodes only have a few connections). Further, the connectivity efficiency E is relatively low, yet the connectivity efficiency has been greatly improved after adding new relationships in *M*_1_. This reflects that there are more reachable paths among the nodes in the network to make the connectivity better.

#### (2) Analysis of network connectivity and path

**Figure 2:**
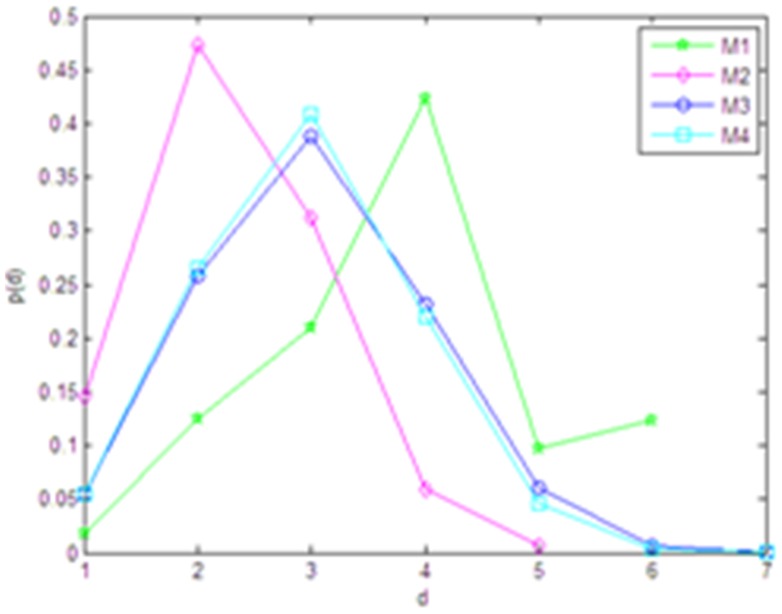
The path length distribution of four types of networks .

The path length distribution of four networks is shown in **Figure 2**. The horizontal ordinate represents the path length of four networks and the ordinate represents the proportion of the path of length d.

#### (3) The characteristic of network degree distribution

The proportion of nodes with different degrees in the networks is shown in **Table 2**. We can see the number of nodes with high degrees increased after adding new interaction relationships in the disease network (**Table 2**).

**Table 2:** The proportion of nodes with different degrees in the networks.

	*Maximum degree*	*k = 1*	*k< = 5*	*k< = 10*	*k< = 15*	*k>20*
*M_1_*	43	45.10%	71.76%	85.88%	92.55%	4.31%
*M_2_*	136	7.06%	20.78%	36.08%	45.10%	49.80
*M_3_*	148	15.88%	42.73%	58.39%	66.67%	30.65%
*M_4_*	148	10.96%	39.82%	55.93%	65.55%	30.65%

#### (4) Analysis of network reliability

In the paper, we analyze the effect of nodes removal on network reliability by randomly removing and determinately removing according to two methods. (a) Randomly removing a certain proportion nodes, removal ratio rises from 0% to 10%. (b) Orderly removing a certain proportion nodes of high degree, removal ratio rises from 0% to 10%.

**Figure 3:**
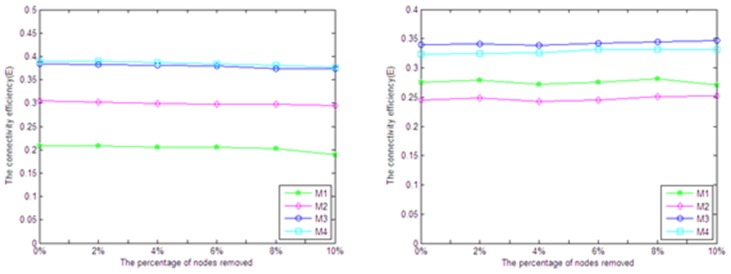
The effect by randomly removing the nodes.

**Figure 4:**
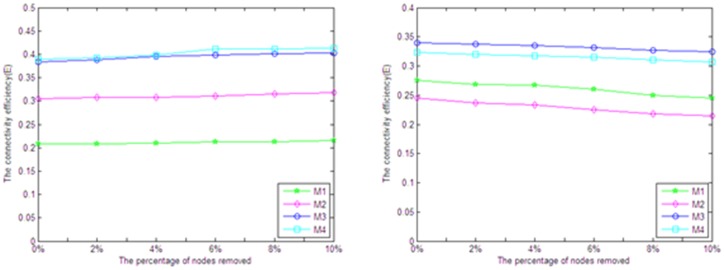
The effect by orderly removing the nodes with high degree.

The effect of these two operations on network connectivity efficiency and standard network structure entropy is shown in **Figure 3** and **Figure 4**. For the random removal, we repeated this operation for 20 times and used their average values in order to avoid the deviation.

It is clear that the random removal of nodes has hardly affected the connectivity efficiency and standard network structure entropy of the networks (**Figure 3**). Similarly, the sequential removal has little effect on the network connectivity efficiency, although the standard network structure entropy decreases slightly (**Figure 4**). In summary, the influence caused by randomly removing and orderly removing on the disease network is very slight, which indicates that the disease network is of great shock resistance and reliability.

### Learning with local and global consistency

The basic idea of LLGC method is to achieve a global stability result through the iteratively outward conductance of local known labels. In the study, all of the drug-disease pairs were first divided into two categories including the pairs with associations and the pairs without associations. Then, we construct a weighted undirected graph using the characteristic values of all the training data based on the manifold and clustering assumption. Nodes in the graph represented all the training examples, and a radial basis function was used to define the weights of edges in the graph.

In order to maximize the effect of the local label diffusion and emphasize the sparse zone of Euclidean distances, we improved the Gauss kernel function:
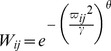
(15)where 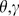 are the variable parameter, 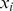 and 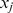 are the feature vector of a drug-disease pair.

We calculate the regularization matrix S of the weight matrix W in order to ensure the convergence of the algorithm:
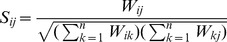
(16)


We make an iterative calculation through the iteration formula:
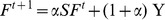
(17)


Where Y is a drug-disease association matrix, the element in the *i_th_* row and *j_th_* column of Y represents whether the *i_th_* disease and the *j_th_* drug is a known drug-disease pair.

This iteration formula adheres to the concept of topological manifold, topologically mapping the neighborhood of points to the high dimensional space. Thus, the diffusion extent of the label of a point in the high dimensional space can be measured by the weights of edges passing through the point in the graph.

### Algorithm analysis

According to the iteration formula, we discovery that 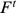 has a limit 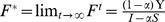 only when 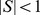. Since S is a regularization matrix, 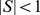 is inevitable, which means 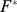 is existent. We can ignore the constant 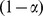 in the calculation process, so we use 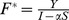 to replace of the iteration formula in order to ensure that there is only truncation error. The energy function of LLGC learning method is described as:
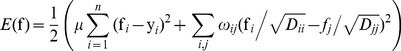
(18)


First, we can get the same iteration limit by calculating the derivative of the energy function.

Then, we set the initial labels of drug-disease pairs:
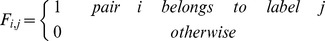
(19)


Where F is a n×2 matrix.

Finally, we analyze the results and label the unknown drug-disease pairs:
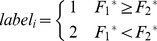
(20)


We infer a drug-disease pair is highly possible to be an associated drug-disease pair if it is labeled with 1.

### Parameter optimization

In this study, three parameters 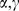 and 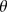 have a significant impact on the performance of the algorithm.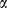 controls the relational degree between each iteration and the initial label information, and the relational degree between each iteration and the label information obtained after iteration. 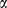 is smaller, the relational degree between each iteration and the initial label information is bigger while the relational degree between each iteration and the label information obtained after iteration is smaller.

**Figure 5:**
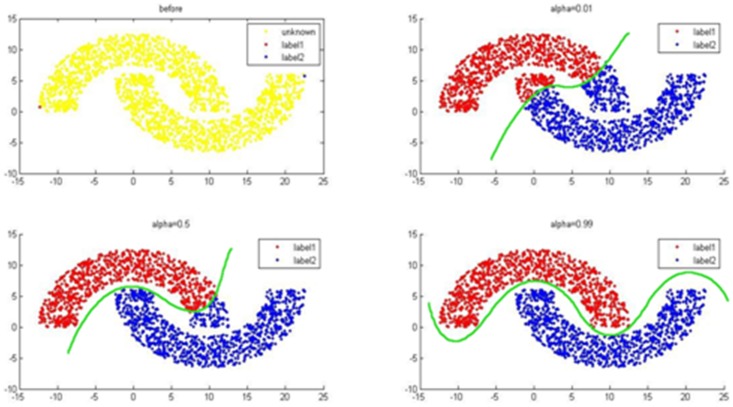
The effect brought by α on the results of classification.


**Figure 6:**
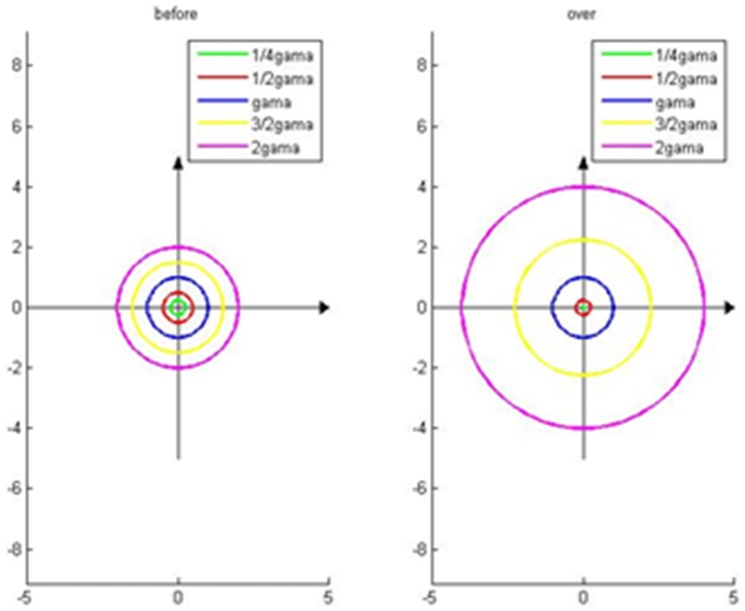
The change of the Euclidean distances between feature vectors.

In the classic two crescent-shaped Toy dataset, we set two instances of known labels for two types of labels. As shown in **Figure 5**, there is a significant influence brought by 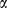 on the results of classification labels. For a dataset with little known labels, local label propagation is more important, meaning that 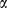 is bigger and the classification result is better. There are little known labels in our dataset, so we set 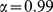 in order to maximize label transfer iteration.

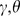
 are the constructional parameters of the weight matrix. 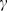 is a reference value. If the Euclidean distance between feature vectors of two drug-disease pairs is smaller than 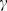, it will be smaller than it is before the transformation. If the Euclidean distance between feature vectors of two drug-disease pairs is bigger than 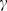, it will be bigger than it is before the transformation. 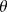 plays a role in amplification and reduction. We take 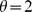 as an example (**Figure 6**). After the transformation, all the distances except the distance with the value equaled to 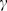 (blue circle) have been amplified or lessened. The distance transform can amplify the local effect of the algorithm, thus make the result better.

**Figure 7:**
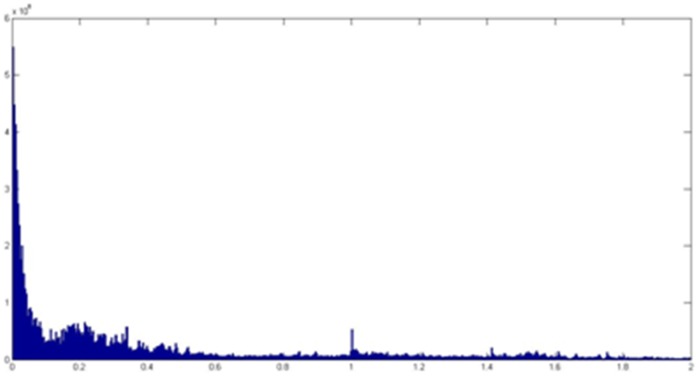
The number of Euclidean distances between feature vectors.

The selection of the value of 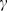 is based on the statistics of the Euclidean distances among feature vectors of all the drug-disease pairs. As shown in **Figure 7**, there is a trough for the number of distances in [0.088, 0.152] which is equivalent to a sparse band for the locations of a vertex relative to the other vertexes in the graph. The semi-supervised learning method based on manifold assumption uses the sparse band like this as the classification boundary. We take the mean value 0.12 of the sparse band as the value of 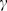, which can make the classification effect more obvious.

### Prediction assessment

**Figure 8:**
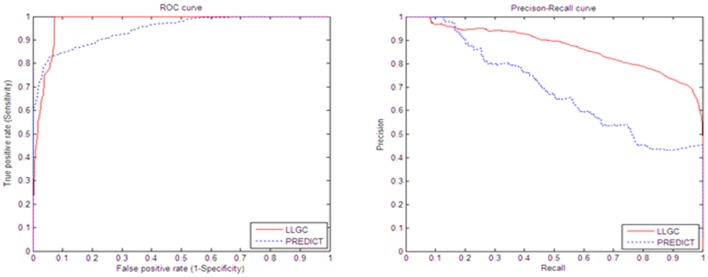
Performance comparison between LLGC and PREDICT method.

PREDICT is a method that can obtain good performance in predicting drug indications by previous references [9]. In order to compare the performance of our method with it, we executed 10-fold cross-validation procedures for 10 times: the dataset of drug-target pairs was divided into 10 subsets, each subset was then taken in turn as a test set and the remaining 9 folds were performed as training set. In each cross-validation, the 548 drug-disease associations are used for training classifier while the remaining 994 unknown drug-disease pairs and 61 drug-disease associations are designated as the testing dataset. The performances of the two methods are evaluated with two quality measures called AUC (Area under the ROC curve) and AUPR (Area under the precision-recall curve). Fi**gure 8** shows the ROC curves and Precision-Recall curves of different methods. As demonstrated in Figure 8, the proposed method obtain the best results with the AUC score of 97.2% and AUPR score of 79.1%, which is increased by 4% on the AUC scores, and 11% on the AUPR scores comparing with PREDICT method.

### Predicted drug-disease associations in the networks

**Figure 9:**
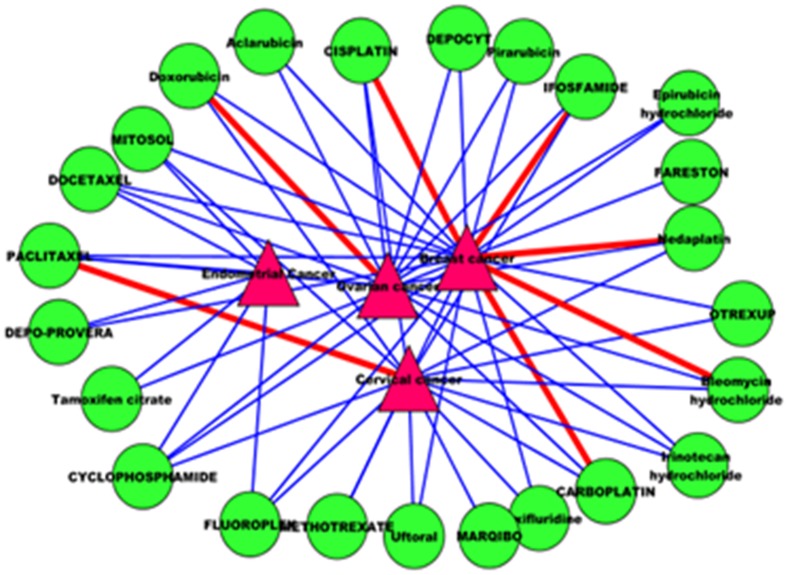
The drug-disease network of breast cancer.

Breast cancer is the most common cancer in women worldwide. It is estimated that more than 1.6 million new cases of breast cancer occurred among women [22]. We focus on the drug-disease network for this important disease in the study. The topological graph of the predicted drug-disease network for breast cancer is demonstrated in Figure 9. We highlighted the predicted drug-disease associations with thick lines. It is shown that breast cancer may have associations with *CISPLATIN*, *IFOSFAMIDE*, *CARBOPLATIN*, *Nedaplatin* and *Bleomycin hydrochloride* (**Figure 9**). Moreover, Doxorubicin and Ovarian cancer, PACLITAXEL and Cervical cancer should be associated drug-disease pairs.

**Figure 10:**
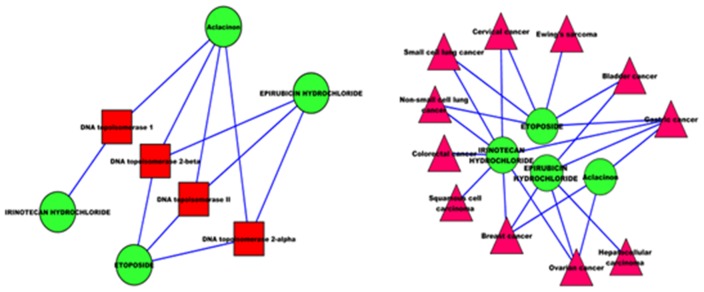
Some drug-target interactions and drug-disease associations in the disease network of breast cancer.

As shown in the left of Figure 10, the drugs like *ETOPOSIDE, Aclacinon, IRINOTECAN HYDROCHLORIDE and EPIRUBICIN HYDROCHLORIDE* have many common targets. So we inferred these drugs may have similar pharmacological functions, thus can be used to treat the same diseases. On the other hand, these drugs have indeed associations with some common diseases in the disease network of breast cancer. Therefore, it is reasonable to encourage further investigation and consider progression to clinical trials for this important disease.
